# RNA interference-based antiviral immune response against the salivary gland hypertrophy virus in *Glossina pallidipes*

**DOI:** 10.1186/s12866-018-1298-1

**Published:** 2018-11-23

**Authors:** Irene K. Meki, Henry M. Kariithi, Andrew G. Parker, Marc J B Vreysen, Vera I D Ros, Just M Vlak, Monique M van Oers, Adly M. M. Abd-Alla

**Affiliations:** 1 Insect Pest Control Laboratory, Joint FAO/IAEA Programme of Nuclear Techniques in Food and Agriculture, International Atomic Energy Agency, Vienna International Centre, P.O. Box 100, 1400 Vienna, Austria; 2 Laboratory of Virology, Wageningen University, 6708 PB Wageningen, The Netherlands; 3 Biotechnology Research Institute, Kenya Agricultural and Livestock Research Organization, P.O Box 57811, Loresho, Nairobi, Kenya

**Keywords:** GpSGHV, RNAi, Sterile insect technique, Glossinidae, Tsetse, Symptomatic and asymptomatic infection, Covert infections

## Abstract

**Background:**

*Glossina pallidipes* salivary gland hypertrophy virus (GpSGHV; *Hytrosaviridae*) is a non-occluded dsDNA virus that specifically infects the adult stages of the hematophagous tsetse flies (*Glossina* species, Diptera: Glossinidae). GpSGHV infections are usually asymptomatic, but unknown factors can result to a switch to acute symptomatic infection, which is characterized by the salivary gland hypertrophy (SGH) syndrome associated with decreased fecundity that can ultimately lead to a colony collapse. It is uncertain how GpSGHV is maintained amongst *Glossina* spp. populations but RNA interference (RNAi) machinery, a conserved antiviral defense in insects, is hypothesized to be amongst the host’s mechanisms to maintain the GpSGHV in asymptomatic (persistent or latent) infection state. Here, we investigated the involvement of RNAi during GpSGHV infections by comparing the expression of three key RNAi machinery genes, *Dicer* (*DCR*), *Argonaute* (*AGO*) and *Drosha*, in artificially virus injected, asymptomatic and symptomatic infected *G. pallidipes* flies compared to PBS injected (controls) individuals. We further assessed the impact of *AGO2* knockdown on virus infection by RT-qPCR quantification of four selected GpSGHV genes, i.e. *odv-e66, dnapol*, maltodextrin glycosyltransferase (a tegument gene) and SGHV091 (a capsid gene).

**Results:**

We show that in response to hemocoelic injections of GpSGHV into *G. pallidipes* flies, increased virus replication was accompanied by significant upregulation of the expression of three RNAi key genes; *AGO1, AGO2* and *DCR2*, and a moderate increase in the expression of *Drosha* post injection compared to the PBS-injected controls. Furthermore, compared to asymptomatically infected individuals, symptomatic flies showed significant downregulation of *AGO1*, *AGO2* and *Drosha*, but a moderate increase in the expression of *DCR2*. Compared to the controls, knockdown of *AGO2* did not have a significant impact on virus infection in the flies as evidenced by unaltered transcript levels of the selected GpSGHV genes.

**Conclusion:**

The upregulation of the expression of the RNAi genes implicate involvement of this machinery in controlling GpSGHV infections and the establishment of symptomatic GpSGHV infections in *Glossina*. These findings provide a strategic foundation to understand GpSGHV infections and to control latent (asymptomatic) infections in *Glossina* spp. and thereby control SGHVs in insect production facilities.

**Electronic supplementary material:**

The online version of this article (10.1186/s12866-018-1298-1) contains supplementary material, which is available to authorized users.

## Background

Tsetse flies (Diptera: Glossinidae) are naturally infected by the *Glossina pallidipes* salivary gland hypertrophy virus (GpSGHV, family *Hytrosaviridae*), a large double-stranded DNA (dsDNA) virus pathogenic specifically to *Glossina* spp., [1, 2]. In *G. pallidipes*, although the majority of cultured and wild tsetse fly species are asymptomatically infected by GpSGHV (low virus titers), some unknown factors can trigger symptomatic infections (high virus titers) [3]. This, in turn, is associated with the occurrence of overt salivary gland hypertrophy (SGH) symptoms [4, 5]. In the mass rearing of *G. pallidipes*, SGH epizootics reduce fly survival and productivity, and have caused the collapse of three colonies; two in the Insect Pest Control Laboratory (IPCL) in Seibersdorf, Austria (in 1987 and 2001), and one in the mass rearing facility in Kality, Ethiopia (in 2012) [6]. These GpSGHV-induced effects have significantly compromised the implementation of the sterile insect technique (SIT), a component of area-wide integrated pest management (AW-IPM) strategies designed for the eradication of *G. pallidipes* from the Southern Rift Valley of Ethiopia [7].

Although it is uncertain how the virus is maintained within tsetse populations in nature and in laboratory colonies, three hypothetical scenarios may account for the maintenance of asymptomatic GpSGHV infection state. The first is a persistent infection whereby the virus remains in specific host cells with low-level production of virions, but without causing substantial cell damage [8]. The second is a latent infection state, during which viral genomes and maybe some viral proteins are present in the infected host cells of certain organs, but without detectable production of infectious viral particles [9]. In the third, the virus can exist in both persistent and latent infection states at the same time, but in different tissues [9]. Persistent infection in the salivary gland (SG) cells is accompanied by a low number of virions (10^2^ viral genome copies/fly) released by asymptomatic flies via saliva during feeding [10]. In addition, detection of viral DNA in other tissues such as the tracheal cells without detectable viral gene transcripts [11] may reflect a latent infection state. In any case, the persistent or latent GpSGHV infection in *G. pallidipes* potentially represents a homeostatic equilibrium between the host’s immune system and the viral escape strategies. Consequently, the viral infection is kept under control (asymptomatic state), but is not completely eliminated from the fly.

Amongst the possible host mechanisms that keep GpSGHV under control is the insect’s RNA interference (RNAi) machinery, which regulates both host and viral gene expression by use of small RNAs that bind to their complementary messenger RNA (mRNA) targets [12, 13]. This hypothesis is based on evidence from various studies indicating that the RNAi machinery is a conserved antiviral defense mechanism for several groups of large dsDNA viruses infecting insects, including the related baculoviruses and nudiviruses, as well as ascoviruses and iridoviruses [14, 15]. RNAi is mediated through three pathways: short interfering RNA (siRNA), microRNA (miRNA) and Piwi-interacting RNA (piRNA) pathways [16]. The siRNAs are processed in the cytoplasm by the ribonuclease III enzyme Dicer-2 (DCR2) from exogenous double stranded (ds) RNAs (e.g. dsRNAs that arise as viral replication intermediates or from overlapping transcripts). These siRNAs destroy (viral or cellular) single-stranded RNAs (ssRNAs) in a sequence-specific manner [17]. The miRNAs, on the other hand, are processed by DCR1 from cellular or viral pre-miRNAs, originating from DNA components of nuclear replicating viruses that are processed by RNase III enzyme Drosha in the nucleus [18]. The miRNAs are then exported to the cytoplasm where they post-transcriptionally regulate cellular or viral protein expression, thereby modulating developmental and physiological processes of the host as well as virus infection [19, 20]. The piRNAs are processed via diverse pathways independent of DCR proteins [21], and are involved in the regulation of cellular genes and activities of transposons [22]. They may also have a role in antiviral strategies, as has been suggested for arboviruses [23]. In the RNAi biogenesis pathways, the siRNAs, miRNAs and piRNAs are loaded into Argonaute proteins 2, 1 and 3 (AGO2, 1 and 3), respectively, that mediate the RNAi by either cleavage or degradation of target RNAs (AGO2), translation repression (AGO1), or epigenetic modifications (AGO3) [24]. However, some of the above-mentioned enzymes may participate in two or more of these pathways. For instance, in *Drosophila melanogaster*, DCR1 is involved in both siRNA and miRNA pathways, while DCR2 is only involved in the siRNA pathway. Additionally, *Drosophila* AGO1 and AGO2 enzymes may participate in both the siRNA and miRNA pathways [25].

The siRNA-mediated RNAi pathway is a potent antiviral immune pathway in insects [21, 26] and is implicated in controlling the replication of RNA and DNA viruses [15, 27–30]. In addition, it has been shown for several viruses that the knockdown of RNAi pathway components leads to increased viral replication. For instance, loss-of-function mutations in *DCR2* enhanced the susceptibility of *Helicoverpa armigera* to infection by *H. armigera* single nucleopolyhedrovirus (HearNPV) [28]. Similar observations were made in *Drosophila* during infection by Flock House virus (FHV), *Drosophila* C virus (DCV), and Sindbis virus (SINV) [31]. In addition to controlling viral replication, the siRNA pathways have also been implicated in establishing persistent virus infection [32]. To establish persistent infection, viral fragments generated during viral DNA genome replication are transcribed or reverse transcribed for RNA viruses and integrated into the host genome. The generated transcripts are processed by DCR2 into virus-derived siRNAs (vsiRNAs); the vsiRNAs are then loaded into AGO2 to mediate specific cleavage of viral mRNAs, leading to persistent infection [32–34]. In the case of GpSGHV infections, the outcome of RNAi-based immune responses would hypothetically be restriction of viral replication and prevention of the development of overt SGH. If this is indeed the case, then virus and host would progress into a stable equilibrium of a persistent or latent infection state, which may account for the widespread chronic asymptomatic GpSGHV infections in many tsetse species, particularly in colonized flies [10].

In the current study, we evaluated whether GpSGHV infection induces an RNAi response in *G. pallidipes* and whether this would downregulate the development of SGH and instead induce a covert infection state (persistent or latent). To accomplish this, we performed comparative analyses of the expression of *AGO*, *DCR* and *Drosha* between artificially (intra-hemocoelic) virus injected and uninfected (PBS injected) individuals, and between asymptomatic and symptomatic infected flies (with overt SGH symptoms). It should be noted that artificial injection of the virus does not result into overt SGH in the same (parental) generations but in the progeny flies [35]. We complemented these bioassays by testing the impact of downregulation of a key component in the siRNA pathway (AGO2), on GpSGHV infection in *G. pallidipes*. The data obtained in this study offer a rationale for similar studies on other *Hytrosaviridae* family members and may open novel strategies to manage SGHVs in insect production facilities.

## Results

### The Argonaute family in *Glossina* species

**Fig. 1:**
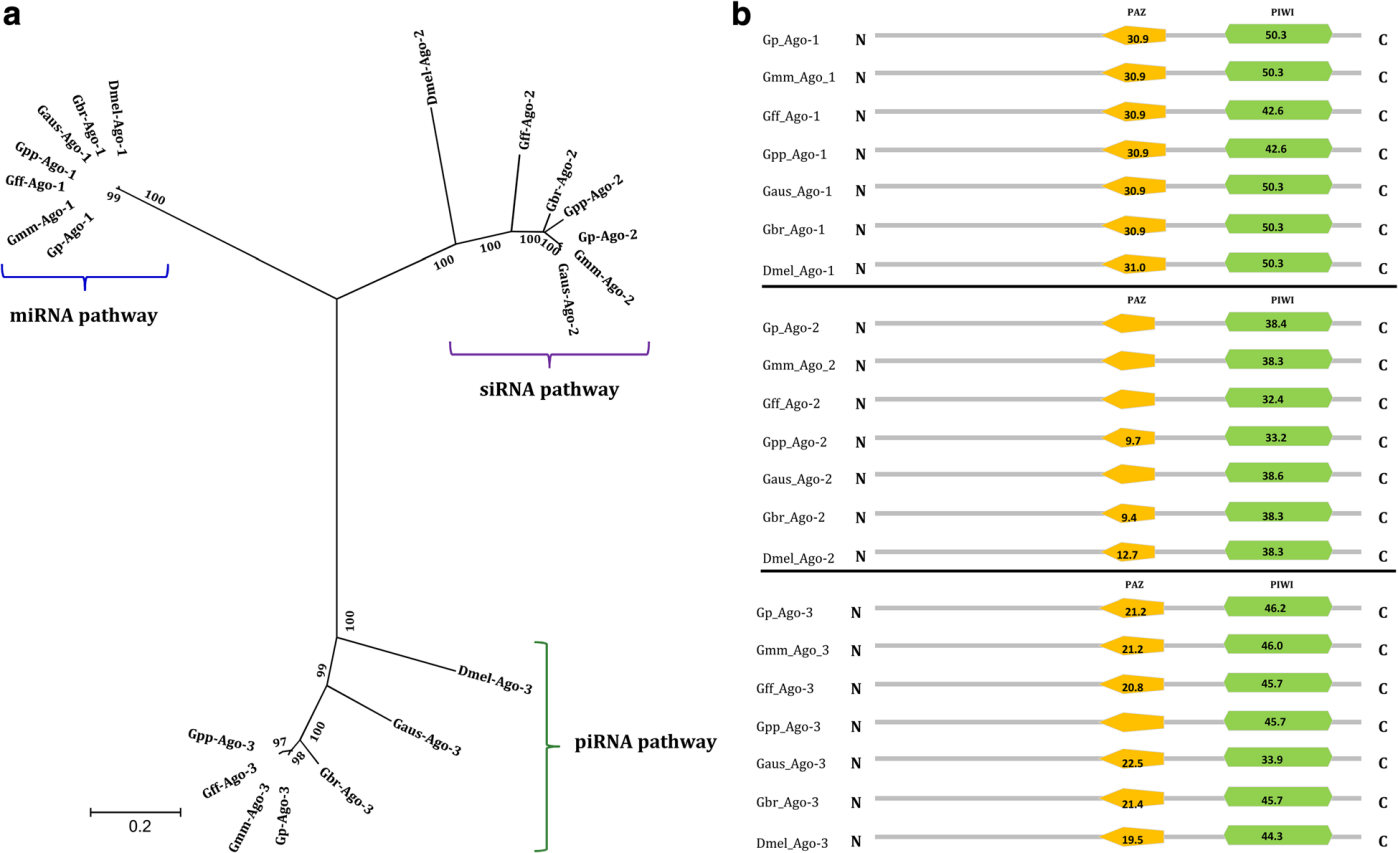
Phylogenetic and domain analysis of Argonaute proteins. **a** Maximum-likelihood based phylogenetic analysis (1000 bootstrap replicates) of Argonaute amino acid sequences of six tsetse species based on full length alignment with *D. melanogaster* as an outgroup. **b** Domain architecture of Argonaute proteins. The numbers on the domains are the scores produced by the ScanProsite search compared to the PROSITE protein domain database. All the tsetse AGO1, AGO2, and AGO3 proteins show similarity in the domain architecture to their orthologs in *D. melanogaster* (Dmel-AGO1, Dmel-AGO2 and Dmel-AGO3), respectively. Abbreviations; AGO (Argonaute), Gp (*G. pallidipes*), Gmm (*G. m. morsitans*), Gff (*G. f. fuscipes*), Gbr (*G. brevipalpis*), Gpp (*G. p. palpalis*), Gaus (*G. austeni*) and Dmel (*D. melanogaster*)

The analyses of the genomes of *G. pallidipes*, *G. m. morsitans*, *G. f. fuscipes*, *G. p. palpalis*, *G. austeni* and *G. brevipalpis* resulted in the identification of AGO 1, 2 and 3 in all these species (See Table 1), key components of the RNA induced silencing complex (RISC); AGOs activate and cleave target mRNA within the RISC complex [36]. The identification of AGOs in the six tsetse species underscores the conservation of the RNAi machinery in *Glossina* species. Phylogenetically, the three AGO proteins segregated into distinct clusters with their orthologs in *D. melanogaster*, which corresponded to the siRNA, microRNA and piRNA pathways of the RNAi machinery (Fig. 1a). The phylogenetic clustering was supported by robust bootstrap values. Additionally, similar to the *D. melanogaster* AGO family proteins, their orthologs in *Glossina* species contained the critical functional domains, i.e. the PAZ domain (for dsRNA binding) and the PIWI domain (executioner of the RNase activity) (Fig. 1b). These results strongly suggest that the three RNAi machinery pathways are functional in *Glossina* spp.

**Table 1:** Accession numbers of *Argonaute*, *Dicer* and *Drosh*a genes of *Glossina* species and *D. melanogaster* found in the VectorBase database

Species	Argonaute 1	Argonaute 2	Argonaute 3	Dicer 1	Dicer 2	Drosha
*G. pallidipes*	GPAI022202-RA	GPAI002659-RA	GPAI022224-RA	–	GPAI041589-RA	GPAI009042-RA
*G. m. morsitans*	GMOY010338-RA	GMOY004940-RA	GMOY010351-RA	GMOY008446-RA	GMOY001890-RA	GMOY008669-RA
*G. fuscipes*	GFUI031750-RA	GFUI006141-RA	GFUI039869-RA	GFUI018989-RA	GFUI024311-RA	GFUI012078-RA
*G. palpalis*	GPPI043499-RA	GPPI035929-RA	GPPI041119-RA	GPPI007107-RA	–	GPPI000118-RA
*G. austeni*	GAUT002476-RA	GAUT035389-RA	GAUT027143-RA	GAUT008865-RA	–	GAUT013637-RA
*G. brevipalpis*	GBRI043708-RA	GBRI017817-RA	GBRI017128-RA	–	GBRI010244-RA	GBRI016708-RA
*D. melanogaster*	NM_166020.2	NM_140518.3	NM_001043162.3	NM_079729.3	NM_079054.5	NM_058088.4

### The dicer family in *Glossina* species

**Fig. 2:**
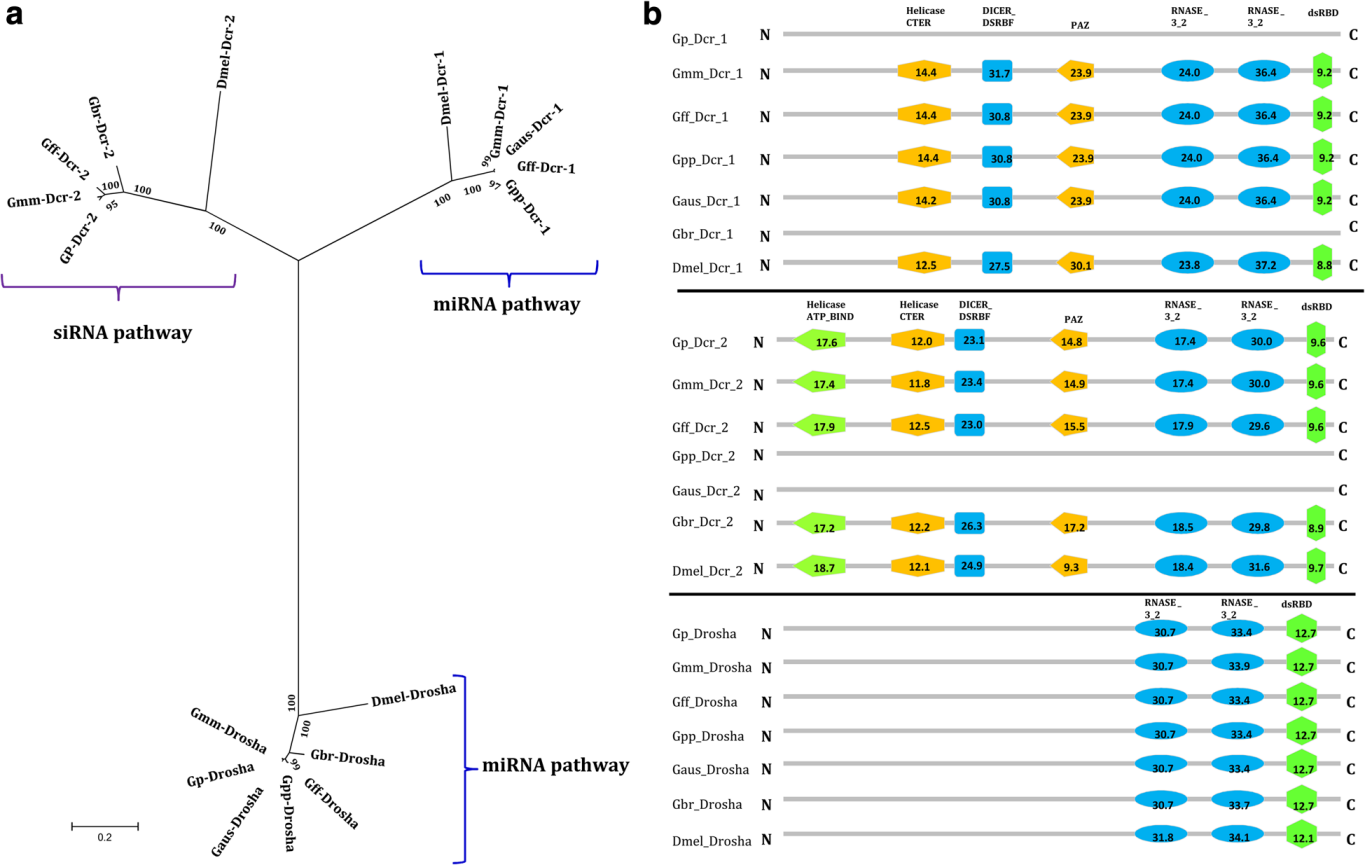
Phylogenetic and domain analysis of Dicer and Drosha proteins. **a** Maximum-likelihood based phylogenetic analysis (1000 bootstrap replicates) of Dicer and Drosha amino acid sequences of six tsetse species based on full length alignment with *D. melanogaster* orthologs as outgroup. **b** Domain architecture of Dicer and Drosha proteins. Some of the tsetse species had either DCR1 or DCR2 proteins, but Drosha was found in all the species. The numbers on the domains are the scores produced by the ScanProsite search compared to the PROSITE protein domain database. All DCR1, DCR2 and Drosha proteins show similarity in the domain architectures to Dmel-DCR1, Dmel-DCR2 and Dmel-Drosha, respectively. Abbreviations; DCR (Dicer), Gp (*G. pallidipes*), Gmm (*G. m. morsitans*), Gff (*G. f. fuscipes*), Gbr (*G. brevipalpis*), Gpp (*G. p. palpalis*), Gaus (*G. austeni*) and Dmel (*D. melanogaster*)

The bioinformatics analysis of the DCR protein family did not result in the identification of the homolog to the *Drosophila* DCR1 protein in *G. pallidipes* and *G. brevipalpis*, but the other *Glossina* species included in this study contained a DCR1 protein homolog (Fig. 2a). However, homologs to the *Drosophila* DCR2 protein were present in both *G. pallidipes* and *G. brevipalpis*, suggesting that DCR2 might be involved in both siRNA and miRNA pathways, at least in these species. However, DCR2 was lacking in *G. p. palpalis* and *G. austeni* (Fig. 2a), implying that in these two species DCR1 might be involved in both siRNA and miRNA pathways. Homologs to both DCR1 and DCR2 were found only in *G. m. morsitans* and *G. f. fuscipes* suggesting that in these species they may be involved in two separate pathways (e.g. miRNA and siRNA) as reported in *Drosophila* [37]. *Drosha* was present in all six tsetse species investigated (Fig. 2a). Our bioinformatics analysis revealed the presence of all the functional motifs in the identified DCR (N-terminal helicases, DCR- dsRBF, PAZ, two C-terminal RNA III, and dsRBD) and *Drosha* (C-terminal RNA III and the dsRBD) protein homologs, which were organized as in their orthologs in *D. melanogaster* (Fig. 2b). Notably, no major differences (phylogenetic and domain architecture) were observed between the DCR1 or DCR2 protein sequences of the tsetse species containing one or both DCR proteins (Fig. 2a and b). The presence of the functional domains in DCR and Drosha homologs could imply the conservation and functionality of both siRNA and miRNA pathways in tsetse. As indicated above, the piRNA pathway is independent of DCR implying that function of this pathway may not be affected by the presence or function of this protein.

### Expression levels of *AGO*, *DCR* and *Drosha* in virus-injected *G. pallidipes*

**Fig. 3:**
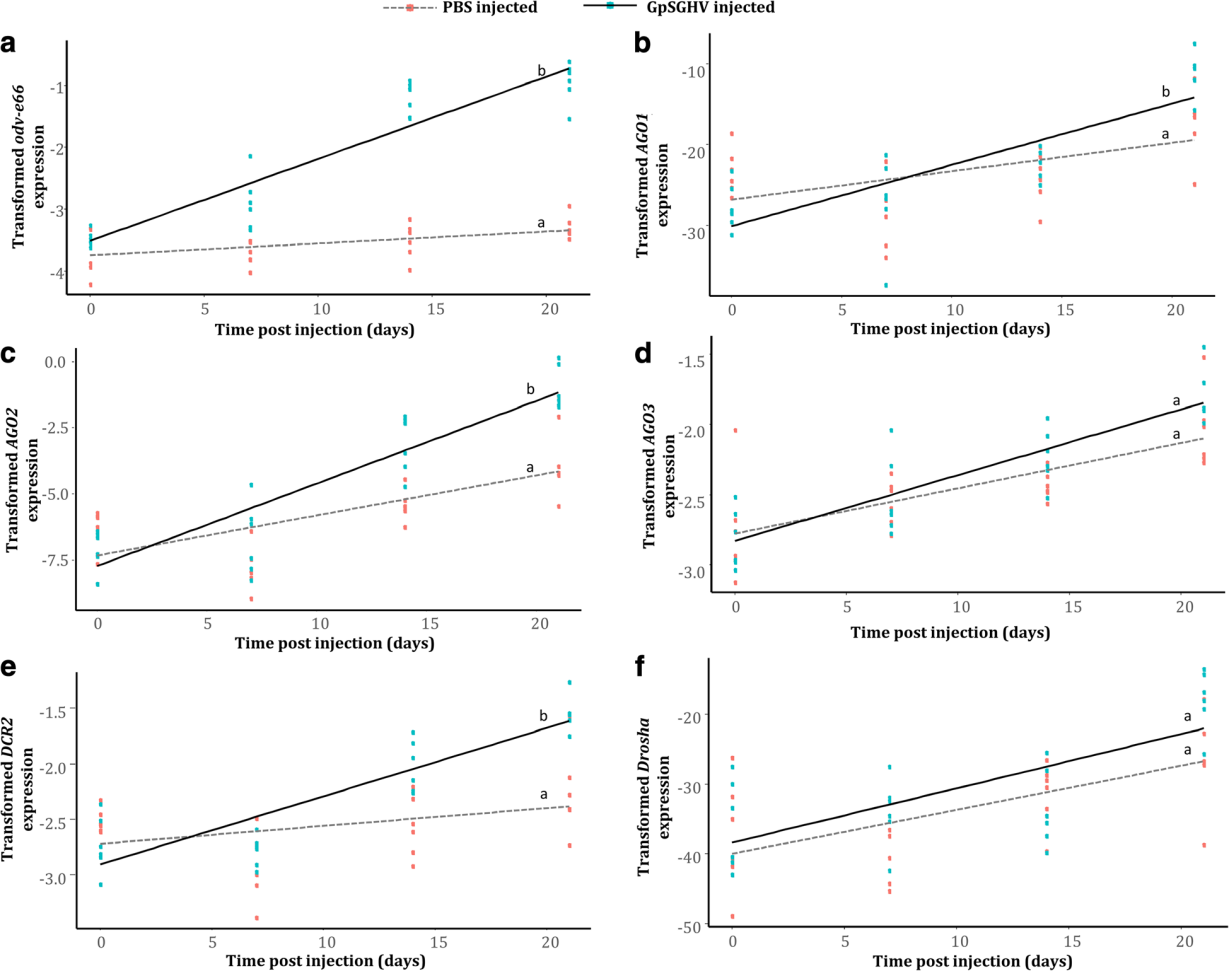
Relative expression of GpSGHV *odv-e66* and RNAi pathway genes post GpSGHV (black line) or PBS (grey dotted line) injection in *G. pallidipes* flies. **a**) GpSGHV *odv-e66*; **b** *AGO1*; **c** *AGO2*; **d** *AGO3*; **e** *DCR2*; and **f** *Drosha*. Gene expression was quantified by RT-qPCR of the RNA extracted from whole fly bodies. Gene expression values were normalized to *β–tubulin* and transformed by the Box-Cox process. The expression levels of *AGO*1, *AGO2* and *Drosh*a were transformed using the lambda (λ) values (Expression^λ^ - 1)/λ), while virus *odv-e66*, *AGO*3 and *DCR2* expressions were log transformed (log(Expression). The results from PBS and virus injection marked with the same lower-case letter do not differ at the 0.05 level

Having identified the core genes involved in the RNAi machinery pathways, we determined whether GpSGHV infection induces an RNAi response. Using *G. pallidipes* as the model species, and due to the absence of *DCR1* in this *Glossina* species (See Fig. 2), we quantified the expression levels of the *AGO* family (*AGO1*, *AGO2* and *AGO3*), *DCR2*, and *Drosha* in flies injected with the virus suspensions compared to the PBS-injected control flies. Additionally, we correlated the expression levels of these RNAi-related genes to the level of virus replication by quantifying the expression levels of GpSGHV *odv-e66*, a conserved and late viral gene. Compared to the control (PBS-injected) fly group, the expression of *odv-e66* increased significantly with time (*t* = 8.657; d.f. = 44; *P* < 0.001) (Fig. 3a), implying active replication and late gene expression of the virus in the injected flies. This increased virus replication was accompanied by a significant increase in the expression level of *AGO1* (*t* = 2.306; d.f. = 44; *P* = 0.026) and *AGO2* (*t* = 3.334; d.f. = 44*; P =* 0.00174) but not *AGO3* (*t* = 1.651; d.f. = 44; *P* = 0.106), of which the *AGO2* (involved in siRNA pathway) was the most upregulated (compare panels B, C and D in Fig. 3). Similarly, to the *AGO* genes *DCR2*, which may be involved in both siRNA and miRNA pathways in *G. pallidipes,* was also found to be significantly upregulated (*t* = 3.968; d.f. = 44; P < 0.001) in response to the virus injection (Fig. 3e). However, unlike *AGO* and *DCR*, the expression levels of *Drosha* showed no significant increase (*t* = 0.601; d.f. = 44; *P* = 0.551) in the virus-injected flies compared to the levels observed in the PBS-injected flies (Fig. 3f). This suggests that *Drosha*, part of the miRNA pathway, is not involved in the immune response against a lytic infection by GpSGHV. Full statistics for each regression are given in Additional file 1**.**

### Expression levels of *AGO*, *DCR* and *Drosha* in symptomatic *G. pallidipes*

**Fig. 4:**
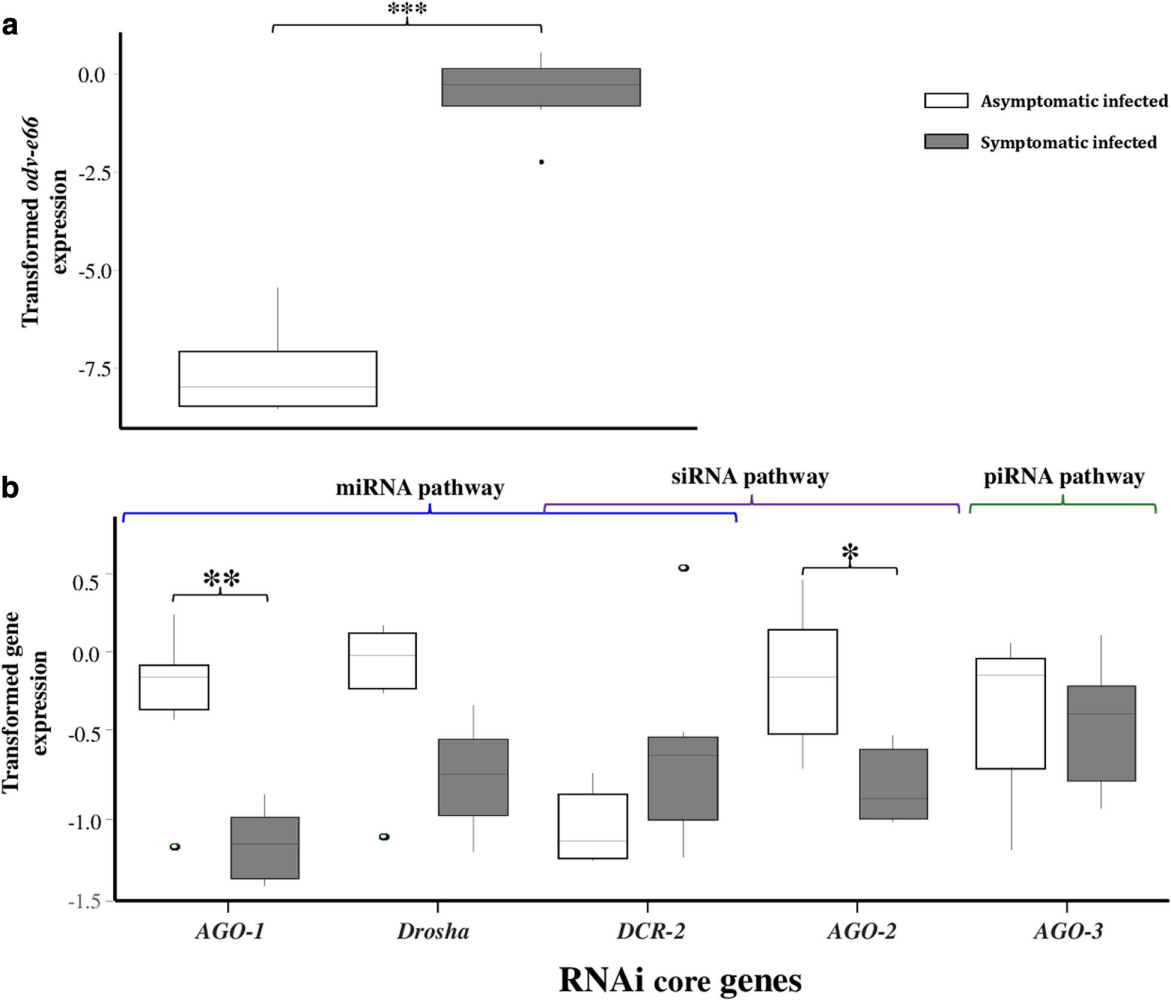
Comparative expression analysis of GpSGHV *odv-e66* and RNAi pathway genes in asymptomatically and symptomatically infected *G. pallidipes* flies. **a** Virus *odv-e66* expression and **b** RNAi genes expression. Gene expression was quantified by RT-qPCR of the RNA extracted from whole fly bodies. Gene expression values were normalized to *β–tubulin* and transformed by the Box-Cox process (log(Expression). The RNAi pathways in which the genes may be involved are also shown. Open boxes = asymptomatic infected; grey boxes = symptomatic infected. Asterisks indicate the statistical significance: ***P < 0.001, **P < 0.01, *P < 0.05

We also compared the expression levels of the *DCR* and *AGO* family genes in the virus-injected *G. pallidipes* flies described above with the expression levels in symptomatic (flies with overt SGH symptoms and high virus titers) and asymptomatically infected individuals (flies with low virus titers) (*t* = 16.72; d.f. = 10; *P* < 0.001) (Fig. 4a). We found that *AGO1* (*t* = − 5.454; d.f. = 10; *P* < 0.001), *AGO2* (*t* = − 3.899; d.f. = 10; *P* = 0.00363) and *Drosha* (*t* = − 3.549; d.f. = 10; *P* = 0.00623) were significantly downregulated in symptomatic *G. pallidipes* flies as compared to asymptomatically infected flies (Fig. 4b). There was no difference in *DCR2* (*t* = 1.318; d.f. = 10; *P* = 0.2202), or *AGO3* (*t* = − 0.858; d.f. = 10; *P* = 0.413) expression between the asymptomatic and symptomatic infected flies (Fig. 4b).

### Effect of *AGO2* knockdown on virus infection in *G. pallidipes*

**Fig. 5:**
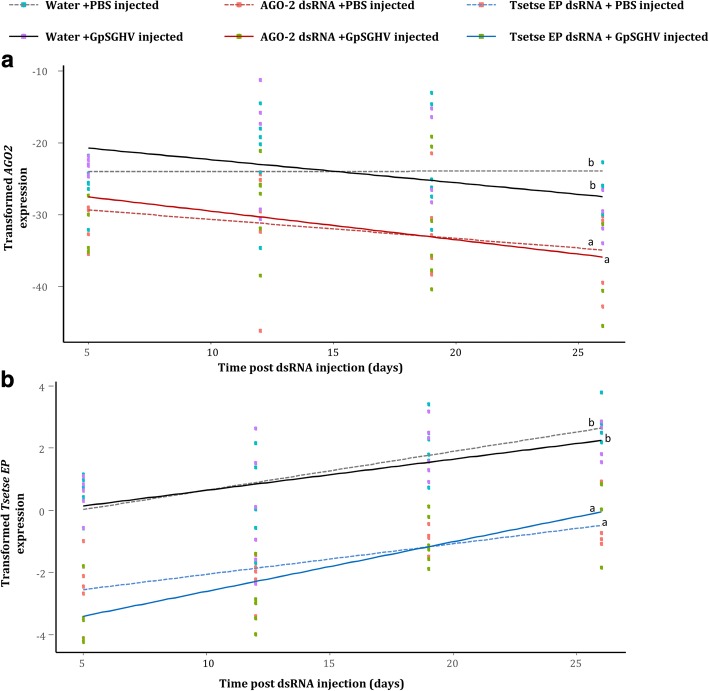
Validation of knockdown of *AGO2* and *tsetse EP* (control) genes in *G. pallidipes*. RT-qPCR expression analysis of: **a**) *AGO2*; and **b**) *tsetse EP* post PBS/virus injection, following *AGO2* and *tsetse EP* dsRNAs injection, respectively, compared to water injected flies (negative controls). Gene expression values were normalized to *β–tubulin* and transformed by the Box-Cox process (Expression^λ^ - 1)/λ). Regression lines marked with the same lower-case letter do not differ at the 0.05 level

**Fig. 6:**
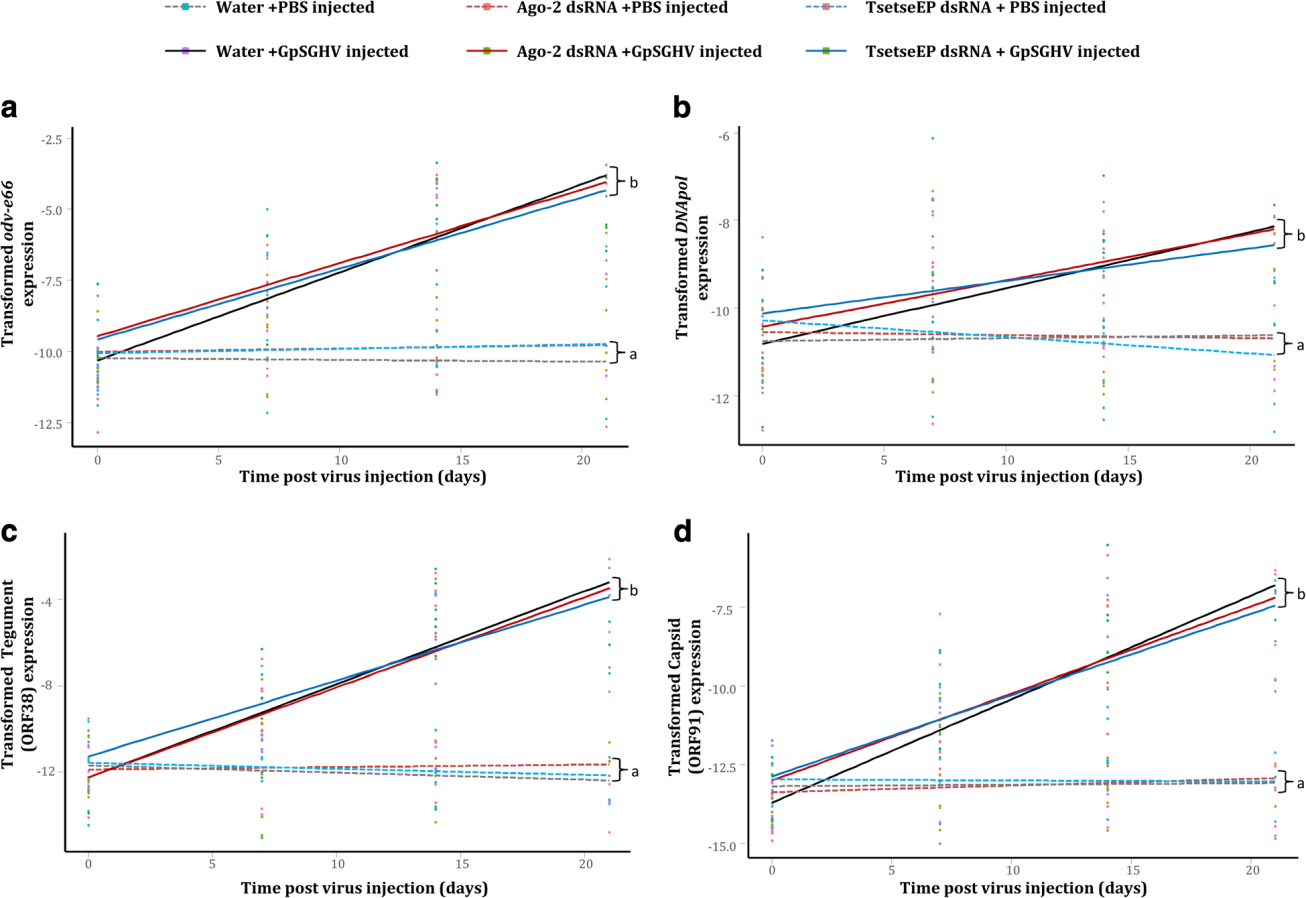
Effect of *AGO2* silencing on selected GpSGHV transcript levels in *G. pallidipes*, following *AGO2* knockdown. RT-qPCR expression analysis of (**a**) GpSGHV *odv-e66* gene, (**b**) GpSGHV *dnapol* gene, (**c**) GpSGHV tegument gene and (**d**) GpSGHV capsid gene post virus injection, following *AGO2* and *tsetse EP* (control) dsRNAs injection. Gene expression values were normalized to *β–tubulin* gene and transformed by the Box-Cox process (log(Expression)). Regression lines marked with the same lower-case letter do not differ at the 0.05 level

We also assessed the impact of *AGO2* knockdown on GpSGHV infection. The *AGO2* gene, which is involved in the siRNA pathway, was chosen for knockdown largely because its expression levels were significantly modulated in both the virus-injected flies (upregulated; see Fig. 3c) and symptomatic infected flies (downregulated; See Fig. 4b). Notably, compared to the flies injected with nuclease-free water, injection of flies with the dsRNAs did not cause any difference in mortality rate. The injection of dsRNAs specific for *AGO2* and *tsetse EP* in addition to PBS or virus injection resulted in a significant decrease in the expression levels of both *AGO2* (AGO2dsRNA/PBS cf. water/PBS: *t* = − 4.265; d.f. = 42; *P* < 0.001, AGO2dsRNA/virus cf. water/virus: *t* = − 3.543 d.f. = 42; P < 0.001) (Fig. 5a) and *tsetse EP* (TsetseEPdsRNA/PBS cf. water/PBS: *t* = − 5.392; d.f. = 40; P < 0.001, TsetseEPdsRNA/virus cf. water/virus: *t* = − 6.798; d.f. = 40; P < 0.001) (Fig. 5b), respectively, compared to the water-injected control flies. We then assessed the effect of *AGO2* knockdown on virus infection by quantifying expression levels of the selected viral genes, *odv-e66*, *DNApol,* SGHV038 and SGHV091. *AGO2* knockdown did not have a significant impact on the transcript levels of any of the selected viral genes; *odv-e66* (*t* = − 1.861; d.f. = 119; *P* = 0.391), *DNApol* (*t* = − 0.422; d.f. = 119; *P* = 0.674), SGHV038 (*t* = − 0.179; d.f. = 119; *P* = 0.858) and SGHV091 (*t* = − 0.877; d.f. = 119; *P* = 0.382) compared to the controls (Fig. 6a, b, c and d). As expected, knockdown of *tsetse EP*, which is not associated with the RNAi machinery, did not affect the expression levels of these selected viral genes. The transformations and parameters for all regression analyses are given in Additional file 1.

## Discussion

In the current study, we have investigated the potential involvement of the RNAi machinery during GpSGHV infections in *G. pallidipes* by quantifying the expression of both the host (*AGO*, *DCR* and *Drosha*) and four viral genes. The finding that AGO and DCR proteins in *Glossina* species contain the functional domains or motifs known to mediate the RNAi response strongly indicate that the RNAi machinery is functional in tsetse, presumably in a similar version as reported in *Drosophila* [38]. It is known that the presence or absence of these functional domains in AGO or DCR proteins affects the efficiency of the RNAi response. For instance, Gu et al. [39] discovered that although AGO2 lacking the PAZ domain interacts with duplex siRNAs, the truncated protein was unable to unwind the siRNAs or eject the passenger RNA strands. The passenger strand should be degraded or ejected from the siRNA duplex to allow the guide strand to be incorporated into the RISC complex and target the mRNA [21]. Moreover, the results from our phylogenetic analysis confirmed that the predicted AGO proteins from the various tsetse species analyzed clustered with those of *Drosophila*.

The presence of only *DCR2* in *G. pallidipes* and *G. brevipalpis*, and only *DCR1* in *G. p. palpalis* and *G. austeni* suggest that in these species only one of the respective proteins is involved in the siRNA and miRNA pathways. In contrast, genes for both DCR1 and DCR2 proteins were found in *G. m. morsitans* and *G. f. fuscipes*. Given that in *Drosophila* DCR1 and DCR2 are involved in the miRNA and siRNA pathways, respectively [37], it is possible that these two proteins are involved in these pathways in these two *Glossina* species as well. Alternatively, they might be involved in both pathways, as suggested for the other *Glossina* species as there were no differences in DCR1 or DCR2 protein sequences of species with one or both DCR proteins, which could be linked to the RNAi pathways. The DCR proteins can be involved in both siRNA and miRNA pathways [24] or be involved in the separate pathways [37]. Taken together, the identification of genes for AGO and DCR proteins may, in principle, be an indication of a robust RNAi silencing response in *Glossina* species [40, 41].

Currently, the mechanism(s) permitting the covert (asymptomatic) GpSGHV infection in *G. pallidipes*, and the reactivation from viral persistence/latency to overt symptomatic infection that is associated with overt SGH symptoms [35], are poorly understood. Our finding of significant upregulation of the expression of *AGO2* in GpSGHV-injected flies suggests that the virus infection induces the host’s siRNA-mediated response, presumably to inhibit the virus infection. Notably *DCR2*, which might be involved in both siRNA and miRNA pathways in *G. pallidipes*, was similarly upregulated during virus infection. The upregulated expression of both *AGO2* and *DCR2* post virus injection in *G. pallidipes*, which are key components in the dsRNA-mediated gene silencing in several insects, suggests a functional RNAi-mediated innate immunity response in *Glossina* species. However, more work is required to elucidate the precise details of this pathway in *Glossina* as well as the involvement of the RNAi machinery in other members of the *Hytrosaviridae* family.

In contrast to the above-mentioned increased levels of *AGO2* and *DCR2* in virus-injected flies, the comparative analysis of the expression of the two genes between asymptomatic and symptomatic infections showed a significant downregulation of the expression of *AGO2*, but insignificant upregulation of *DCR2* in the flies exhibiting diagnostic SGH symptoms. The high expression of siRNA pathway genes in the virus-injected flies suggests a tight control of the virus via the RNAi response during asymptomatic GpSGHV infections. However, during symptomatic infections as evidenced by increased virus titers, the siRNA pathway may be compromised (as supported by the low expression of *AGO2*) enabling the virus to escape the RNAi-mediated innate immunity, thereby increasing virus titers and in turn causing the detectable SGH symptoms. A similar outcome has been documented in the case of the African malaria mosquito, *Anopheles gambiae*, where dsRNA-mediated silencing of *AGO2*, which functions in conjunction with *DCR2* in this mosquito, resulted in increased O’nyong-nyong virus (ONNV) viral loads [42]. It should be noted that, due to its involvement in both the siRNA and miRNA pathways, *DCR2* was not considered a suitable candidate for the knockdown assays in this study. Therefore, we assessed the potential involvement of dsRNA-mediated gene silencing (siRNA pathway) in *G. pallidipes* by knockdown of *AGO2.*

Several examples of the function of RNAi in insects have been clearly demonstrated, including for species from the orders Diptera, Dictyoptera, Isoptera, Hymenoptera and Orthoptera [43, 44]. In the case of tsetse flies, Walshe et al., [45] showed that micro-injection of dsRNA into 6–8 day-old *G. m. morsitans* flies could persistently silence expression of *tsetse EP,* a gene that is demonstrated to protect the fly against establishment of trypanosome infections in the midgut [46]. In the current study, the knockdown of *AGO2* in *G. pallidipes* did not alter the transcript levels of the selected GpSGHV genes implying that *AGO2* knockdown had no effect on the GpSGHV infection. These findings are in contrast to previous results; for instance in *Drosophila melanogaster* in which flies that were deficient in the DCR2 protein showed increased susceptibility to infection by members of three different RNA virus families; i.e. FHV (*Nodaviridae*), DCV (*Dicistrovirida*e), and SINV (*Togavirida*e) [31]. In general, many studies have led to the conclusion that flies that contain mutations in genes that encode components of the siRNA pathway (including *DCR2* and *AGO2*) or the Janus kinase/signal transducers and activators of transcription (JAK-STAT) pathway, are not only more sensitive to infection by several viruses, but also harbor higher viral titers than their wild-type counterparts. The JAK-STAT pathway is also a conserved insect innate immune antiviral response [13, 26, 31, 38, 47]. In another report, *D. melanogaster* defective for the *AGO2* were found to be hypersensitive to infections by DCV, an infection which also supported a 1000-fold increased production of progeny virus [47].

The results obtained from the current study showed that reduction of RNAi efficiency in *G. pallidipes* did not cause a significant impact on the fly immunocompetence. Notably, in addition to the RNAi pathway investigated in this study, the JAK-STAT, immune deficiency (imd) and Toll immune pathways are also involved in elimination of viruses [48–50]. The presence of these innate immune pathways may indicate that following the interruption of the RNAi pathway in *G. pallidipes* flies in our study alternative antiviral pathways in these flies were able to control the virus infections.

## Conclusions

Given the high diversity of pathogens, their elimination by host organisms is challenging and therefore many organisms, including insects, employ multiple mechanisms to deal with them. The RNAi machinery, particularly the siRNA pathway, plays a central role in insects by specifically recognizing and eliminating invading pathogens and other invasive elements such as transposons. This study has elucidated important milestones in the infection of GpSGHV in *Glossina* spp. We detected RNAi key genes in all the analyzed *Glossina* species, which may indicate a functional antiviral role of RNAi machinery in tsetse flies. In *G. pallidipes*, the model *Glossina* species in this study, the siRNA pathway genes *AGO2* and *DCR2* were upregulated during virus infection, which confirmed the involvement of the RNAi response in the flies’ defense against GpSGHV. We also noted that in *G. pallidipes*, the siRNA pathway was compromised during symptomatic infection as evidenced by the low expression of *AGO2*. However, although knockdown of *AGO2* in *G. pallidipes* did not have an impact on virus infection, it would be worthwhile to further investigate the long-term effects of the gene knockdowns on GpSGHV transmission and the expression of SGH symptoms in F_1_ progeny produced by the parents with the knockdown. The F_1_ generation is of interest since induction of SGH symptoms does not occur in the parental generation of flies that are intra-hemocoelically injected with the virus but are observed in the subsequent F_1_ generation produced by injected mothers [35]. It may therefore be important to determine whether the dsRNA-mediated gene silencing is a heritable trait, as has been demonstrated in other studies [51]. The next question would be to determine which factors (exogenous or endogenous) trigger the transition from the asymptomatic to symptomatic state in colonized and wild flies. Together, the approach and results of the current study offer a starting point for further investigations into *Hytrosaviridae* family members and may open novel strategies to control SGHVs in insect production facilities.

## Methods

### Tsetse fly and virus injections

The *G. pallidipes* flies used in this study were obtained from the colony maintained at the Joint FAO/IAEA IPCL, Seibersdorf, Austria. *G pallidipes* was selected for this study because, unlike other species, it shows both asymptomatic and symptomatic virus infections [3]. The experimental *G. pallidipes* flies were maintained in controlled insectaria with 70–80% relative humidity, 24 ± 1 °C temperature and 12 h photo-phase. The flies were fed for 10–15 min, 3 times per week on defibrinated bovine blood using an in vitro membrane feeding system [52]. The virus inoculum was prepared from a one pair of hypertrophied salivary glands (with overt SGH symptoms) dissected from *G. pallidipes* male flies; viral titers in the gland homogenates (in PBS) were estimated by qPCR as described previously [3, 35]. The experimental flies were injected with 2 μl of the virus inoculum estimated to contain ~ 10^6^ virus genome copies per μl.

### Identification of core RNAi gene orthologs in *Glossina*

To determine whether the *G. pallidipes* genome contains the key RNAi pathway genes, *AGO*, *DCR* and *Drosha* sequences were retrieved from the VectorBase database [53] using as query sequences the annotated homologous gene sequences of *G. morsitans morsitans* [54] (BLASTp; e-value ≤10^− 2^). To determine the conservation of RNAi in *Glossina* species, sequences of these core genes for *G. fuscipes fuscipes*, *G. palpalis palpalis*, *G. austeni* and *G. brevipalpis* were similarly retrieved from VectorBase. The functional domain architecture of the retrieved *AGO*, *DCR* and *Drosha* sequences was analyzed using the ScanProsite tool [55]. Homologous search of the retrieved sequences was performed to determine the relatedness of these sequences with those of *D. melanogaster* for which RNAi mechanisms and pathways have been demonstrated. Multiple alignments of protein-coding loci of the identified gene sequences were performed in BioEdit [56]. Phylogenetic analysis was performed with MEGA6 using default settings for Maximum-Likelihood (ML) based on the General Time Reversible model with gamma distributed substitution rates with 1000 bootstrap replications [57].

### Analysis of the expression of core RNAi genes in virus-injected *G. pallidipes* by RT-qPCR

To investigate the impact of GpSGHV on the core RNAi genes in *G. pallidipes*, two groups of teneral flies (newly-eclosed and non-fed; 50 females and 50 males per group) were injected with either the virus inoculum as described above, or phosphate buffered saline (PBS) as control. Following the injections, four females and four males were sampled within 1 h post injection and at 7, 14 and 21 days post injection (dpi). Total RNA was extracted from individual whole bodies of the sampled flies using Trizol reagent (Invitrogen, Paisley UK) according to the manufacturer’s instructions. Contaminating DNA was removed from the extracted RNA by treating the samples with DNase 1 (Invitrogen, Paisley, UK), after which the concentration of the RNA was quantified using a Nanodrop ND-1000 spectrophotometer (Thermo Fisher Scientific, Wilmington, DE). Complementary DNAs (cDNAs) were synthesised using the SuperScript® III Reverse Transcriptase kit (Invitrogen, Paisley UK) following the manufacturer’s instructions. The iQ SYBR green supermix (Bio-Rad laboratories, Hercules, CA) was used for RT-qPCR analysis. The viral infection was assessed by quantifying the expression of GpSGHV *odv-e66*, a conserved, late viral gene (highly expressed upon viral genome replication), followed by expression analysis of *AGO*, *DCR* and *Drosha* transcripts, using the PCR cycling conditions: 95 °C for 3 min, followed by forty cycles of 95 °C for 10 s, 60 °C for 1 min, then 95 °C for 1 min and 55 °C for 1 min, using the primers shown in Table 2. The tsetse housekeeping gene *β-tubulin* was used to normalize gene expression.

**Table 2:** Sequences of the primers used in synthesis of dsRNAs and for expression analysis by RT-qPCR

Target gene	Primer name	Primer sequence (nt) – Primers are listed 5′- to − 3′	Reference
A Primers for dsRNAs synthesis			
*Argonaute 2*	AGO-2 T7-F	TAATACGACTCACTATAGGGGTCTTAGCATCCAACAACCA	This study
	AGO-2 T7-R	TAATACGACTCACTATAGGGTGTCTATGCCGCACTCTTTC	
*Tsetse EP*	TseEPT7-F	TAATACGACTCACTATAGGGCTACGATAAATATGTCCCTC​TAAT	Modified from [45]
	TseEPT7-F	TAATACGACTCACTATAGGGATCGGGCAAACCCTCAAC	
A Primers for q-RT-PCR			
*Argonaute 1*	AGO-1qPCR-F	CAACTGCTCGTTCGGCTCCA	This study
	AGO-1qPCR-R	GGCAAAACTCGTCCTCTTACTTCCA	
*Argonaute 2*	AGO-2qPCR-F	CGTTGGATGATGGCACAAAGATG	This study
	AGO-2qPCR-R	GCTGCCTGATGTGATGCAATTC	
*Argonaute 3*	AGO-3qPCR-F	GCACAACTAGCAGAGATGACAGATAC	This study
	AGO-3qPCR-R	TGCAGGGCAATCTTTTGGACAAT	
*Dicer 2*	DCR-2qPCR-F	GTAGAGCGAAGATACACGGCTAAA	This study
	DCR-2qPCR-R	CACCATAAATTGCGGCCTAATGAC	
*Drosha*	DroshaqPCR-F	TCAAAACCAAGGACAGAGCGGA	This study
	DroshaqPCR-R	GCAAACGGGGAAAAAGGCAAAC	
*Tsetse EP*	TseEPqPCR-F	ACCGTTCGTTCGCTTTACTAC	Modified from [45]
	TseEPqPCR-R	ACCAGCAGCCGTTTGACTTTC	
GpSGHV (*odv-e66*)	GpSGHVqPCR-F	CAAATGATCCGTCGTGGTAGAA	[3]
	GpSGHVqPCR-F	AAGCCGATTATGTCATGGAAGG	
GpSGHV (Maltodextrin glycosyltransferase, tegument protein)	GpSGHV32F	ACGCTGAACTAAATTATCGTCATCTACACG	This study
	GpSGHV31R	CACAGAATCGTCATCATCATCATCTACAGA	
GpSGHV (capsid protein)	GpSGHV92F	TATATTGTAATCCACGACCGGAAACTGAAC	This study
	GpSGHV91R	TCGGTAGGCGTGAATGAACGTTTT	
*β-Tubulin* (tsetse)	Tse-TubqPCR-F	GATGGTCAAGTGCGATCCT	[66]
	Tse-TubqPCR-R	TGAGAACTCGCCTTCTTCC	

### Differential expression of RNAi genes in asymptomatic and symptomatic flies

To determine the differential expression of *AGO*, *DCR* and *Drosha* between asymptomatic and symptomatic infected flies, 10-day old F_1_ progeny flies produced by virus injected mothers were screened under a stereo microscope for the occurence of diagnostic SGH symptoms. Total RNA was extracted from whole bodies of 8 asymptomatic and 8 symptomatic infected flies (4 females and 4 males). The viral infection was estimated by quantification of the GpSGHV *odv-e66* transcripts, followed by expression analysis of the three genes as described above. These expression analyses were replicated three times (biological replicates).

### Design of dsRNA constructs and prediction of off-targets

The optimal regions on the *AGO2* mRNA for the synthesis of dsRNA constructs were determined by siRNA design software (default setting) [58], which uses three predictive steps; (i) selection of functional siRNA sequence, (ii) selection of siRNA sequence with reduced off-target effects and (iii) elimination of near-perfect matched off-target genes. The identification of off-targets was performed by BLAST (BLASTn; e-value ≤10^− 2^) search of VectorBase. Following identification of the siRNAs, primers for dsRNAs synthesis were designed to flank the most effective siRNAs based on the above-described steps and a T7 promoter sequence added on each primer (See Additional files 2 and 3).

### Synthesis of dsRNAs

To generate dsRNA to knockdown *AGO2*, total DNA was isolated from *G. pallidipes* using the Qiagen DNeasy Blood and Tissue kit (QIAGEN Inc., Valencia, CA). The extracted DNA was subsequently used to produce T7 tailed PCR amplicons of *AGO2* using primers designed to contain 5′-T7 promotor sequences (See Table 2). These primers allowed dsRNAs transcription using the Hiscribe T7 Quick high yield RNA synthesis kit (New England Biolabs, UK) according to the manufacturer’s instructions. Template DNA was removed from the transcription reaction by DNase treatment, as described in the transcription kit. The synthesized dsRNAs were purified using MEGAclear columns (Ambion, ThermoFisher Scientific, USA) and eluted in 50 μl nuclease free water. The *tsetse EP* gene, an immune response gene with extensive glutamic acid-proline dipeptide repeats, that has been successfully knocked down in tsetse, [45, 46] was used to assess the efficiency of the knockdown treatment (i.e. by measuring the expression of the *tsetse EP* gene).

### Injections of flies with GpSGHV and dsRNAs

To investigate the impact of *AGO2* knockdown on GpSGHV infection, teneral *G. pallidipes* flies were divided into three groups each consisting of 40 females and 40 males and offered one blood meal. After 48 h, two of three groups were injected with 4 μl of either *AGO-2* or *tsetse EP* dsRNAs (2.5 μg/μl dsRNA in RNase free water) (See the procedure in Additional file 4). The selection of this dsRNA dose was optimized for effective knockdown based on previous bioassays on dsRNA-mediated gene knockdown in tsetse flies [45, 46]. The third group of flies (an additional negative control) was injected with RNase-free water. For the injections, flies were anaesthetized by chilling (~ 5 min) on ice, and subsequently injected in the dorsolateral surface of the thorax. Five days after the dsRNAs/RNase-free water injections, half of the injected flies (20 females and 20 males) from each group were injected with 2 μl of the virus suspension as described above, while the other half were injected with PBS. This time point (i.e. 5 days post dsRNA injection) was selected because successful gene knockdown in tsetse has been shown to occur after ~ 3 dpi [46], implying that the 5 dpi in our case ensured that the virus was injected after successful knockdown. To monitor the impact of *AGO2* knockdown on GpSGHV infection, 3 females and 3 males were sampled from each of the above described treatment groups at 1 h post injection, and at 7, 14 and 21 dpi. The samples were stored at − 20 °C until further analysis as described below.

### Analysis of the impacts of *AGO2* knockdown on GpSGHV replication

To assess the effect of *AGO2* knockdown of GpSGHV replication, total RNA was extracted and cDNA synthesized as described above from the frozen fly samples collected from different time points post dsRNA and virus injection. The efficiency of gene knockdowns was assessed by quantifying (by RT-qPCR) *AGO2* and *tsetse EP* gene transcripts using the qPCR primer sets listed in Table 2. The impact of *AGO2* knockdown on GpSGHV infection was assessed by RT-qPCR quantification of mRNA transcripts of the selected conserved GpSGHV genes; the per os infectivity *odv-e66* (SGHV005) gene, *DNApol* (SGHV079) gene involved in DNA replication, a tegument gene (SGHV038) and capsid gene (SGHV091) [59, 60]. Note that a clear correlation between the GpSGHV*odv-e66* gene transcripts and the total virus copy numbers has been previously reported [3], which may demonstrate the impact of *AGO2* knockdown on virus replication.

### Statistical analysis

All quantitative RT-qPCR results were representative of at least three independent biological experiments, each with three technical replicates. Statistical differences in the expression of the above described host and viral genes between the different treatments and the controls were performed with RStudio v1.0.143 [61] (R v3.4.0 [62]) using the packages lattice v0.20–35 [63] and MASS v7.3.47 [64] The obtained data were visualized using the ggplot2 v2.2.1 package [65] available within the RStudio platform. Data was checked for normality and transformed where necessary using the Box-Cox routine. The data was log transformed where the confidence interval of lambda includes 0 and transformed with (x^λ^-1)/λ in other cases. T-tests were used for the comparison of RT-qPCR data.

## Additional files

Additional file 1: Regression parameters applied in the statistical analysis. For each regression line (column 2) found in the figures (column 1), the confidence interval of lambda values (column 3) obtained based on the Box-Cox routine to determine the method of transformation (column 4) of the data, the intercept values (column 5), the t value (column 6) the *P* values (column 7) and the degrees of freedom (D.F) in column 8. (DOCX 23 kb)

Additional file 2: Result of *AGO2* dsRNA design and off-target prediction. A). *AGO2* mRNA sequence; the primers flanking the knockdown sequence are highlighted in cyan, predicted siRNAs in grey and qPCR primers in yellow. (B) Shows a graphical view of effective siRNAs candidates. (TIF 9175 kb)

Additional file 3: Result of *tsetse EP* dsRNA design and off-target prediction. A). *Tsetse EP* mRNA sequence; the primers flanking knockdown sequence are highlighted in cyan, predicted siRNAs in grey and qPCR primers in yellow. (B) Shows a graphical view of effective siRNAs candidates. (TIF 8491 kb)

Additional file 4: Step by step procedure to determine the effect of Knock-down of *AGO2* on virus infection in *G. pallidipes*. Teneral *G. pallidipes* flies were collected and offered one blood meal. 48 h later the flies were divided onto three groups and injected with 4 μl *AGO2* dsRNAs, *tsetse EP* dsRNAs or RNase-free water. Five days later, each of the 3 groups was divided into two and each injected with either 2 μl of the virus suspension or with PBS. Three females and 3 males were sampled from each of the described treatments at 1 h post injection, and at 7, 14 and 21 days post injection to determine the effect of *AGO2* knockdown on virus infection. (TIF 1030 kb)

## Abbreviations

AGO: Argonaute
AW-IPM: area-wide integrated pest management
DCR: Dicer
DCV: *Drosophila* C virus
DsRNA: double stranded RNA
FHV: Flock house virus
GpSGHV: *Glossina pallidipes* salivary gland hypertrophy virus
HearNPV: *Helicoverpa armigera* single nucleopolyhedrovirus
Imd: immune deficiency
IPCL: Insect Pest Control Laboratory
JAK-STAT: Janus kinase/signal transducers and activators of transcription
miRNA: microRNA
piRNA: Piwi-interacting RNA
RISC: RNA induced silencing complex
RNAi: RNA interference
SGH: salivary gland hypertrophy
SINV: Sindbis virus
siRNA: short interfering RNA
vsiRNAs: virus-derived siRNAs
