# Supplementary material for: RNA interference-based antiviral immune response against the salivary gland hypertrophy virus in Glossina pallidipes
Source: BMC Microbiol. 2018 Nov 23;18(Suppl 1):170. doi: 10.1186/s12866-018-1298-1 (PMC6251114; doi:10.1186/s12866-018-1298-1)
Supplement: Supplementary file 1 — Regression parameters applied in the statistical analysis. For each regression line (column 2) found in the figures (column 1), the confidence interval of lambda values (column 3) obtained based on the Box-Cox routine to determine the method of transformation (column 4) of the data, the intercept values (column 5), the t value (column 6) the P values (column 7) and the degrees of freedom (D.F) in column 8. (DOCX 23 kb) [file 12866_2018_1298_MOESM1_ESM.docx]

**Additional file 4:** Regression parameters applied in the statistical analysis. For each regression line (column 2) found in the figures (column 1), the confidence interval of lambda values (column 3) obtained based on the Box-Cox routine to determine the method of transformation (column 4) of the data, the intercept values (column 5), the t value (column 6) the P values (column 7) and the degrees of freedom (D.F) in column 8.

| **Figure** | **Regression line** | **Lambda value** | **Transformation** | **Intercept value** | **t value** | **P value** | **D.F** |
| --- | --- | --- | --- | --- | --- | --- | --- |
| 3A | PBS injected | -0.0606 | Log(X) | -3.74481 | 8.657 | 4.7e-11 | 44 |
|  | GpSGHV injected |  |  |  |  |  |  |
| 3B | PBS injected | -0.4646 | (X^λ^ - 1)/λ | -26.8421 | 2.306 | 0.02591 | 44 |
|  | GpSGHV injected |  |  |  |  |  |  |
| 3C | PBS injected | -0.3030 | (X^λ^ - 1)/λ | -7.32758 | 3.334 | 0.00174 | 44 |
|  | GpSGHV injected |  |  |  |  |  |  |
| 3D | PBS injected | -0.1414 | Log(X) | -2.77941 | 1.651 | 0.106 | 44 |
|  | GpSGHV injected |  |  |  |  |  |  |
| 3E | PBS injected | 0.02020 | Log(X) | -2.72081 | 3.968 | 0.000264 | 44 |
|  | GpSGHV injected |  |  |  |  |  |  |
| 3F | PBS injected | -0.6667 | (X^λ^ - 1)/λ | -40.0065 | 0.601 | 0.55097 | 44 |
|  | GpSGHV injected |  |  |  |  |  |  |
| 5A | Water +PBS injected | -0.5858 | Log(X) | -4.62802 | -4.265 | 0.000111 | 42 |
|  | Ago-2 dsRNA +PBS injected |  |  |  |  |  |  |
|  | Water +GpSGHV injected | -0.7474 | (X^λ^ - 1)/λ | -4.59147 | -3.543 | 0.000984 | 42 |
|  | Ago-2 dsRNA +GpSGHV injected |  |  |  |  |  |  |
| 5B | Water +PBS injected | 0.1414 | (X^λ^ - 1)/λ | -4.24996 | -5.392 | 3.37e-06 | 40 |
|  | TsetseEP dsRNA + PBS injected |  |  |  |  |  |  |
|  | Water +GpSGHV injected | 0.1414 | (X^λ^ - 1)/λ | -6.51110 | -6.798 | 3.59e-08 | 40 |
|  | TsetseEP dsRNA + GpSGHV injected |  |  |  |  |  |  |
| 6A | Water +GpSGHV injected | 0.02020 | Log(X) | -10.31518 | Reference | | 119 |
|  | Water +PBS injected |  |  |  | -5.237 | 7.14e-07 |  |
|  | Ago-2 dsRNA +PBS injected |  |  |  | -4.948 | 2.49e-06 |  |
|  | TsetseEP dsRNA + PBS injected |  |  |  | -4.889 | 3.20e-06 |  |
|  | Ago-2 dsRNA +GpSGHV injected |  |  |  | -0.861 | 0.391 |  |
|  | TsetseEP dsRNA + GpSGHV injected |  |  |  | -0.995 | 0.322 |  |
| 6B | Water +GpSGHV injected | -0.0606 | Log(X) | -10.82670 | Reference | | 119 |
|  | Water +PBS injected |  |  |  | -2.391 | 0.018393 |  |
|  | Ago-2 dsRNA +PBS injected |  |  |  | -2.654 | 0.009048 |  |
|  | TsetseEP dsRNA + PBS injected |  |  |  | -3.259 | 0.001459 |  |
|  | Ago-2 dsRNA +GpSGHV injected |  |  |  | -0.422 | 0.673527 |  |
|  | TsetseEP dsRNA + GpSGHV injected |  |  |  | -1.047 | 0.297225 |  |
| 6C | Water +GpSGHV injected | -0.0606 | Log(X) | -12.241639 | Reference | | 119 |
|  | Water +PBS injected |  |  |  | -5.910 | 3.33e-08 |  |
|  | Ago-2 dsRNA +PBS injected |  |  |  | -5.351 | 4.31e-07 |  |
|  | TsetseEP dsRNA + PBS injected |  |  |  | -5.849 | 4.44e-08 |  |
|  | Ago-2 dsRNA +GpSGHV injected |  |  |  | -0.179 | 0.858 |  |
|  | TsetseEP dsRNA + GpSGHV injected |  |  |  | -1.018 | 0.311 |  |
| 6D | Water +GpSGHV injected | -0.1010 | Log(X) | -13.71663 | Reference | | 119 |
|  | Water +PBS injected |  |  |  | -5.441 | 2.88e-07 |  |
|  | Ago-2 dsRNA +PBS injected |  |  |  | -5.163 | 9.89e-07 |  |
|  | TsetseEP dsRNA + PBS injected |  |  |  | -5.578 | 1.55e-07 |  |
|  | Ago-2 dsRNA +GpSGHV injected |  |  |  | -0.877 | 0.382 |  |
|  | TsetseEP dsRNA + GpSGHV injected |  |  |  | -1.180 | 0.240 |  |
